# Diabetic Muscle Infarction: A Rare Cause of Acute Limb Pain in Dialysis Patients

**DOI:** 10.1155/2013/931523

**Published:** 2013-05-07

**Authors:** G. De Vlieger, B. Bammens, F. Claus, R. Vos, K. Claes

**Affiliations:** ^1^Department of Nephrology and Renal Transplantation, University Hospitals Leuven, Herestraat 49, 3000 Leuven, Belgium; ^2^Laboratory of Nephrology, Department of Microbiology and Imunology, KU Leuven, Herestraat 49, 3000 Leuven, Belgium; ^3^Department of Radiology, University Hospitals Leuven, Herestraat 49, 3000 Leuven, Belgium; ^4^Respiratory Division, University of Leuven and Department of Clinical Respiratory Medicine, Catholic University of Leuven, Herestraat 49, 3000 Leuven, Belgium

## Abstract

Diabetic muscle infarction is a rare microangiopathic complication occurring in patients with advanced diabetes mellitus. Diabetic patients with chronic kidney disease stage Vd are prone to develop this complication. The presenting symptom is a localized painful swelling of the affected limb. Symptoms usually resolve spontaneously during the following weeks, but frequent relapse can occur and in some cases swelling may lead to compartment syndrome. Biochemical blood analyses show an elevated C-reactive protein, but creatine kinase is often normal. Diagnosis can be made on clinical presentation and imaging, with magnetic resonance imaging as the gold standard. Histology is often not contributive. Treatment consists of rest, analgesics, rigorous glycemic control and low-dose aspirin. Severe cases of compartment syndrome require fasciotomy. In the current paper, we present two diabetic patients with cystic fibrosis, who are treated with automated peritoneal dialysis and suffered from episodic lower limb infarction. We subsequently review 48 episodes of diabetic muscle infarction previously reported in the literature in patients with end-stage renal disease.

## 1. Introduction

Diabetic muscle infarction (DMInf) is a rare microangiopathic complication in patients with advanced diabetes mellitus (DM). Patients having terminal diabetic nephropathy are prone to develop DMInf and nearly one-fourth of DMInf, patients receive renal replacement treatment [1]. Consequently, nephrologists are likely to be increasingly confronted with this disease entity. 

## 2. Case Reports

**Figure 1:**
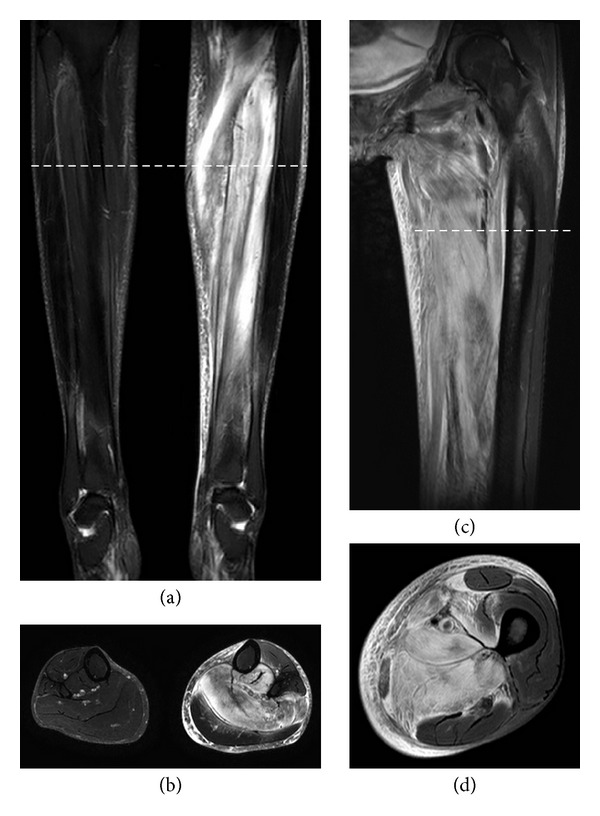
T2 weighted images with coronal (a, c) and axial (b, d) views for the 2 patients. The level of the axial views are indicated on the coronal images by the dashed lines. Images (a) and (b) show diffuse edema in the calf muscles of the left lower limb of the first patient, as illustrated by the hyperintense signal of the muscular tissue (note the normal low signal intensity of the muscles in the right leg). Images (c) and (d) show similar changes in the adductor muscles of the left upper leg of the second patient.

*Case *1. A 27-year-old woman with cystic fibrosis started insulin treatment at the age of 11. When she was 16 years old, she received bilateral lung transplantation (SSLTx). Her immune-suppressive therapy consisted of tacrolimus and steroids. At the age of 24, she developed chronic kidney disease stage V (CKD-Vd) for which peritoneal dialysis (PD) was started. Two years later, she presented with acute pain in the right calf. Biochemical evaluation showed an elevated creatine kinase (CK 218 U/L) and C-reactive protein (CRP 97 mg/L). HbA1c was 5.8%. Ultrasound and computed tomography (CT) showed diffuse muscular and subcutaneous edema of the affected calf. Muscular biopsy demonstrated muscular atrophy, macrophages, and myophagia. The symptoms resolved within four weeks. There was a new onset of pain in the left calf 18 months later. CK was normal, but CRP levels were elevated (215 mg/L). HbA1c was 7.2%. The clinical and biochemic characteristics are shown in Table 1. Magnetic resonance imaging (MRI) showed an infarction in the soleus muscle (Figures 1(a) and 1(b)). During the following days, pain increased and a compartment syndrome was diagnosed by pressure measurement. A fasciotomy was performed, and low-dose aspirin was started with subsequent resolution of the complaints in the following two months.

*Case *2. A 35-years-old woman with cystic fibrosis received SSLTx at the age of 21. Redo SSLTx because of chronic lung allograft rejection was performed six years later. Immunosuppressive treatment consisted of tacrolimus and steroids. DM was diagnosed at the age of 29, and subcutaneous insulin treatment was initiated one year later. When she was 34 years old, PD was started. Six months later, she was admitted because of pain in the left thigh. The pain was present during rest and increased during exercise. Clinical examination revealed a swollen and painful left upper leg with absence of deep venous thrombosis (DVT) on ultrasound. MRI showed muscle infarction with central necrosis in the adductors, medial vastus muscle, and sartorius muscle (Figures 1(c) and 1(d)). CRP was elevated (94 mg/L), but CK's were repeatedly normal. The DM was poorly controlled with an HbA1c of 8.7%. Low-dose aspirin was started, and the symptoms resolved after six weeks. Six months later, she was readmitted because of severe pain in the right thigh. Biochemical examination showed an increased CRP of 104 mg/L, the CK was within the normal range, and HbA1c was 7,5%. MRI showed edema in the left pectineus muscle, external obturator, and adductor muscles. Analgesia was started with spontaneous resolution. 

## 3. Discussion

DMInf was first described in 1965 by Angervall and Stener [2]. It is a rare microangiopathic complication in patients with advanced DM. The typical presentation is an acute onset of severe pain at rest, exacerbated by movement. Clinical examination usually reveals a hard and warm palpable swelling of the affected limb, and ultrasound is negative for DVT. 

In a review of 166 episodes in 115 diabetic patients by Trujillo-Santos, the mean age at presentation of DMInf was 42.6 years, and the mean duration of DM was 14.3 years with 59% of the patients suffering from DM type 1 [17]. A female predominance (61,5%) was observed, and associated microangiopathic complications included nephropathy, retinopathy, and neuropathy in 71.1, 56.6, and 54.5%, respectively [17]. We described two patients with cystic fibrosis who evolved to CKD-V after SSLTx. Both patients were treated with PD and were at high risk for the development of DMInf due to multiple risk factors: DM type 1, administration of calcineurin inhibitors and prothrombotic status due to uremia. Although there is no evidence of increased thrombogenic risk in cystic fibrosis, lung transplantation is clearly associated with a hypercoagulability [18] and an increased risk for DVT and pulmonary embolism [19]. The two presented cases were treated with PD, but it is not clear whether PD is associated with a higher risk to develop DMInf compared to hemodialysis. Previous studies report a higher concentration of advanced glycation end products (AGEs) that play an important role in the pathogenesis of vasculopathy [20]. Moreover, conventional PD fluids contain glucose degradation products (GDP) that cause an increased production of AGEs [20]. In both of our patients, conventional solutions were used from the start of PD treatment, and they were switched to a low GDP solution after a few months because of infusion pain. It is tempting to speculate whether the use of low GDP solution from the start could have avoided the DMInf.

We reviewed the literature and discussed 48 episodes of DMInf in 30 patients with CKD-Vd [3–16]. Mean and median age at time of diagnosis of DM was 28.1 and 30.5 years respectively, and DMInf occurred at a mean and median age of 46.0 and 49.0 years. In our review, there was a slight predominance for males (56.8%). Recurrent muscle infarctions were reported in one third of the patients. Data comparing the different dialysis modalities were not available. The most affected muscle groups were located in the upper leg (68.8%) and lower leg (22.9%) only few (8.3%) were located in the upper limb. Other diabetic complications were retinopathy (80.8%), neuropathy (69.2%), and macrovasculopathy (34.6%). 

Symptoms of DMInf generally resolve spontaneously after an average of six weeks [21]. However, evolution to compartment syndrome might occur, warranting meticulous followup and prompt fasciotomy in severe cases. Biochemical evaluation shows increased CRP, and CK's may be elevated during the early presentation. Later in the disease course, CK's appear to be normal in most cases [22]. Consequently, laboratory tests are not accurate in diagnosing DMInf. Sonographic examination typically demonstrates focal or diffuse muscle edema of the affected limb and is in particular useful to exclude deep venous thrombosis [23]. CT can detect muscular swelling and vascular calcifications, but is not sensitive to detect early muscle ischemia (10). MRI has a high tissue contrast resolution and is the ideal imaging modality to depict muscular edema and pathological changes in the fatty intramuscular septa, subfascial tissue, and the subcutis. The radiologic differential diagnosis of diabetic muscle infarction includes an intramuscular abscess, myositis, and necrotizing fasciitis, and muscle biopsy might be necessary to obtain a final diagnosis [24]. However, excisional biopsy and surgical debridement in these patients can be complicated by delayed wound healing, hematoma, infection, nerve palsy, heterotopic calcification, and need for blood transfusion [21]. In the majority of patients, the diagnosis is based on the clinical presentation and MRI findings, hence reducing the need for biopsy and its associated complications. Evolution to a compartment syndrome has been described in a few cases. Patients suffering from infarction of the thigh muscles are less prone to develop a compartment syndrome due to the larger osteofascial envelope [25]. 

Overall, treatment consists of rest, analgesia, and rigorous glycemic control. Low-dose aspirin shortens the recovery time to 5.5 weeks as compared to 8.1 weeks in those treated with rest and analgesia alone. Surgical resection of the infarcted muscles increases the recovery period to 13 weeks [26] and is not a first-choice option. Physical therapy may be beneficial, but it has also been reported that it causes worsening or relapse [27]. The short-term prognosis of the muscle infarction is good since it is often a self-limiting disease. If a compartment syndrome is suspected, pressure measurement should immediately be performed with subsequently fasciotomy if the diagnosis is confirmed. Recurrence in the same or contralateral limb has been reported in 50 to 62% of patients [6]. However, the overall survival of these patients seems to be comparable with the prognosis after myocardial infarction with a 55% 1-year survival rate in one report [28]. Due to the increase overall cardiovascular mortality in CKD-V patients, the prognosis is probably worse in our studied population. Consequently, treatment with low-dose aspirin, rigorous glycemic control, and control of the serum phosphorus concentration is of paramount importance.

In summary, we discussed the clinical presentation of two diabetic patients with recurrent episodes of DMInf after initiation of peritoneal dialysis. Clinical and sonographic examination might suggest the diagnosis and is useful to rule out the presence of DVT. Contrast-enhanced MRI is the imaging modality of choice to confirm the diagnosis of muscle infarction and to screen for patients at risk to develop compartment syndrome.

## Figures and Tables

**Table 1:** Overview of reviewed cases in the literature.

Ref	Age	Gender	Type DM	Age at onset	DM complications other than CKD	RRT	Laboratory			Affected muscles
							HbA1c	CRP (mg/dL)	CK (U/L)	
	24	Female	DM I	11	Retinopathy	APD	5.8	97	218	(1) Left vastus lateralis (2) Left calf

[3]	29	Female	DM I	9	Neuropathy	SPK	5	—	160	Right gastrocnemius

[4]	29	Female	DM I	8	Retinopathy, neuropathy	SPK		—	17	Right gastrocnemius and soleus

[5]	31	Male	DM I	—	Retinopathy, coronaropathy	IHD	—	—	—	Left vastus lateralis

[6]	31	Female	DM I	16	Retinopathy, peripheral vascular disease	IHD	12.4	—	649	(1) Right vastus lateralis (2) Left thigh (3) Left calf

[7]	32	Male	DM I	20	Retinopathy, neuropathy, and peripheral vascular disease	IHD	—	—	350	Left vastus muscles

[4]	39	Female	DM I	9	Retinopathy, neuropathy, and ischemic cardiac disease	SPK	5.0	—	21	Right deltoid muscle

[4]	33	Female	DM I	7	Retinopathy, neuropathy	SPK	5.6	—	Normal	Left biceps brachii

	35	Female	DM I	30		APD	8.7	94.2	97	(1) Left vastus medialis and intermedius (2) Right adductors

[4]	39	Female	DM I	9	Retinopathy, neuropathy	SPK, IHD	5.3	—	83	Right thigh adductors

[8]	49	Male	DM I	—	Retinopathy, neuropathy	CAPD, IDH	7.9	214	—	(1) Left thigh (2) Right vastus medialis (3) Left thigh (4) Right calf

[8]	51	Male	DM I	41	—	IHD	—	216	381	(1) Left buttock (2) Right semitendinosus

[9]	35	Male	DM II	27	Retinopathy, coronary disease	CAPD	—	—	556	Right vastus medialis

[5]	39	Male	DM II	39	Retinopathy, neuropathy	IHD	—	—	Elevated	Left gastrocnemius

[8]	40	Male	DM II	25	Retinopathy, neuropathy, and peripheral vascular disease	CAPD, IHD	—	—	—	Left rectus femoris

[7]	44	Female	DM II	41	Retinopathy, neuropathy	IHD	—	—	—	(1) Right thigh (2) Left calf

[9]	49	Female	DM II	34	Retinopathy, neuropathy	CAPD, IHD	—	—	Normal	(1) Left calf (2) Left gluteal region (3) Right thigh

[10]	49	Male	DM II	—	Heart failure	IHD	—	35	289	Right rectus femoris

[8]	49	Female	DM II	—	Neuropathy	IHD	—	300	692	Anterior right thigh

[11]	51	Male	DM II	—	—	IHD	—	—	Normal	Left thigh

[12]	55	Male	DM II	35	—	IHD	—	—	463	Left vastus lateralis

[13]	55	Male	DM II	31	Retinopathy, neuropathy	CAPD	—	105	Normal	(1) Left thigh (2) Right thigh (3) Right thigh

[5]	56	Female	DM II	—	Retinopathy, neuropathy	CAPD	—	—	Elevated	Left quadriceps

[5]	58	Male	DM II	—	Retinopathy, neuropathy, and peripheral vascular disease	IHD	—	70	Normal	Bilateral quadriceps
[8]	61	Male	DM II	49	Neuropathy, and ischemic heart disease	IHD	8	136	23	Right gastrocnemius and soleus

[14]	61	Female	DM II	45	Retinopathy, neuropathy	IHD	6.7	—	1066	(1) Left vastus medialis (2) Right thigh (3) Right calf

[15]	62	Male	DM II	52	Retinopathy	IHD	6.2	—	69	Left triceps and forearm

[15]	63	Male	DM II	38	Retinopathy, neuropathy	IHD	9.5	—	483	Right brachioradialis

[8]	63	Male	DM II	43	Retinopathy, neuropathy, ischemic heart disease, and stroke	IHD	—	361	409	(1) Right vastus medialis and lateralis (2) Left thigh (3) Left hamstrings (4) Left hamstrings

[16]	67	Male	DM II	—	—	KTx	—	—	—	Right gastrocnemius and soleus

DM: diabetes mellitus, CKD: chronic kidney disease, RRT: renal replacement therapy, APD: automated peritoneal dialysis, SPK: simultaneous pancreas kidney transplantation, IHD: intermittent hemodialysis, CAPD: continuous ambulatory peritoneal dialysis, KTx: kidney transplantation, CRP: C-reactive protein, and CK: creatine kinase.
